# Adjuvant radiotherapy versus observation following gross total resection for atypical meningioma: a systematic review and meta-analysis

**DOI:** 10.1186/s13014-021-01759-9

**Published:** 2021-02-17

**Authors:** Se-Woong Chun, Kyung Min Kim, Min-Sung Kim, Ho Kang, Yun-Sik Dho, Youngbeom Seo, Jin Wook Kim, Yong Hwy Kim, Chul-Kee Park

**Affiliations:** 1 Department of Rehabilitation Medicine, Gyeongsang National University Changwon Hospital, Gyeongsang National University School of Medicine, Changwon, Korea; 2 Department of Neurosurgery, Seoul National University Hospital, Seoul National University College of Medicine, 101 Daehak-ro, Jongno-gu, Seoul, 03080 Korea; 3 Department of Neurosurgery, Chungbuk National University Hospital, Chungbuk National University College of Medicine, Cheongju, Korea; 4 Department of Neurosurgery, Yeungnam University Hospital, Yeungnam University College of Medicine, Daegu, Korea

**Keywords:** Atypical meningioma, Gross total resection, Adjuvant, Radiotherapy, Postoperative

## Abstract

**Background:**

The impact of adjuvant radiotherapy (RT) on atypical meningioma (AM) underwent a gross total resection (GTR) remains unclear, showing conflicting results from various studies. The objective of this study was to perform an updated meta-analysis for observational studies to determine the effect of adjuvant RT after GTR on local recurrence and survival outcomes compared to observation after GTR.

**Methods:**

PubMed, Embase, and Web of Science were searched to identify comparative studies that reported outcomes of adjuvant RT versus observation for AM patients after GTR. Local recurrence rate, progression-free survival (PFS), overall survival (OS), and toxicities related to RT were considered as outcomes of interest. Differences between two cohorts were estimated by calculating odds ratios (OR) for LR rate and hazard ratios (HR) for survival outcomes with 95% confidence intervals (CIs) for meta-analysis, using R version 4.0.3 software. Included studies were appraised with the Risk of Bias Assessment tool for Non-Randomized Studies. Outcome ratios were combined with the Mantel–Haenszel method and the inverse variance-weighted method, appropriately.

**Results:**

Data from 30 studies involving 2904 patients (adjuvant RT: n = 737; observation: n = 2167) were eventually included. Significant reduction of local recurrence rate was seen in the adjuvant RT cohort compare to that in the observation cohort (OR 0.50; 95% CI 0.36–0.68; *p* < 0.0001). Pooled HRs of PFS at 1-year, 3-year, 5-year, and > 5-year revealed that adjuvant RT was superior to observation. There was no significant difference in OS between the two cohorts during any period. Most toxicities were tolerable with grade 1 or 2. There was no documented grade 5 toxicity.

**Conclusions:**

For AM patients who underwent GTR, evidence suggested that adjuvant RT could potentially decrease local recurrence and improve PFS better than observation.

## Introduction

Since the implementation of the 2007 World Health Organization (WHO) grading classification, the first to consider brain invasion as a diagnostic criterion of atypical meningioma (AM), the proportion of AM in newly diagnosed meningioma has increased from less than 10% to approximately 20–35% [1–5]. Traditionally, maximal surgical resection has been considered as the most important factor for predicting the prognosis of AM. However, even a after gross total resection (GTR), recurrence can occur in a substantial portion of patients because of its unpredictable behavior and heterogeneity [6–8]. The recurrence of AM has been associated with significant morbidity and increased mortality. Thus, effort to reduce recurrence should be prioritized in the management of AM patients.

It is widely accepted that adjuvant radiotherapy (RT) can affect favorable prognosis of AM patients after a subtotal resection (STR). However, the impact of RT on AM patients after a GTR remains controversial. Several retrospective studies have investigated the effect of adjuvant RT compared to observation after GTR, showing inconsistent results most likely due to a small sample size of each study, especially in the cohort that receives adjuvant RT following GTR [6, 9–16]. Recently, two multi-cohorts non-randomized phase II observational studies have reported a potential benefit of local control with adjuvant RT after GTR in AM patients [17, 18]. However, neither study was designed to directly compare adjuvant RT versus observation in AM patients with GTR. Two randomized phase III trial, ROAM/EORTC-1308 (Radiotherapy versus Observation following surgical resection of Atypical Meningioma/European Organization for Research and Treatment of Cancer-1308) and NRG-BN003 (Observation Versus Irradiation for a Gross Totally Resected Grade II Meningioma) are currently underway to investigate whether adjuvant RT is superior to observation for reducing the recurrence of AM after GTR [19, 20]. Until results of these randomized trials are known, it is necessary to synthetically analyze outcomes of previously reported studies for deciding an optimal treatment strategy for AM patients after GTR.

The primary aim of the current study was to compare local recurrence and survival outcomes between adjuvant RT and observation cohorts of AM patients after GTR. To achieve this aim, we conducted an updated systematic review and meta-analysis for relevant clinical observational studies with a comparative design.

## Materials and methods

### Search strategy and study selection criteria

In accordance with the PRISMA (Preferred Reporting Items for Systematic Reviews and Meta-Analyses) statement [21], we performed a meta-analysis of clinical studies that investigated the effect of adjuvant RT after GTR on AM. A thorough search for eligible studies in electronic databases of PubMed, Embase, and Web of Science from inception to August 10th, 2020 was conducted by two independent researchers (M.S.K and S.W.C). Search strategies utilized for each database are presented in Additional file 1: Table S1. Search results were screened by scanning abstracts using the following exclusion criteria: case report, technical note, review, letter or conference abstract; duplicate study; single cohort study; article dedicated to adjuvant radiosurgery (e.g., gamma knife surgery, cyberknife, stereotactic linear accelerator-based radiosurgery), proton or carbon ion radiotherapy, or brachytherapy; and article not about the population of interest (e.g., clear cell meningioma, chordoid meningioma). After all retrieved studies were reviewed, reference lists of reviews were also screened for qualifying studies. Only articles in English were considered since Morrison et al. reported no significant difference in pooled effects from the use of language restrictions in systematic review-based meta-analysis in medicine [22]. No limitation was set on the date of publication. Any discrepancies between the two reviewers (M.S.K and S.W.C) were resolved by discussion.

### Inclusion criteria, data extraction, and quality assessment

The goal of the search was to find articles that met the following inclusion criteria: (1) articles that described two distinctive cohort groups of AM patients who received either GTR only (observation cohort) or adjuvant RT after GTR (adjuvant RT cohort); and (2) articles that reported the outcome of local recurrence rate, progression-free survival (PFS), and/or overall survival (OS) for each cohort. Studies with a non-homogeneous design that reported outcomes for AM with STR or malignant meningioma were included if they separately reported outcomes according to whether adjuvant RT was performed or not after GTR for AM. We excluded articles that included patients with extracranial AM, salvageable RT for recurrent AM after GTR, and neurofibromatosis.

The following data were extracted from selected studies: author of study, year of publication, study design, country that the study was conducted, period of research, version of adopted WHO classification, definition of GTR in each study, total number of each cohort, recurrence rate or number, PFS, OS, and any complication associated with adjuvant RT, if feasible. Toxicities of each study were reevaluated for grading using Common Terminology Criteria for Adverse Events (CTCAE) version 5.0.

The methodological quality for an individual article was assessed using the Risk of Bias Assessment tool for Non-Randomized Studies (RoBANS) [23]. Two authors (M.S.K and S.W.C) performed a quality appraisal of each study independently. A consensus was reached by discussion for any discrepancies among reviewers.

### Statistical analyses

The primary outcome was direct comparisons of local recurrence rate, PFS, and OS in the adjuvant RT and the observation cohort after GTR for AM. For analysis of local recurrence, numbers of recurred patients in the adjuvant RT and the observation cohort were identified. Odds ratios (OR) and 95% confidence intervals (CIs) were combined using the Mantel–Haenszel statistical method. Hazard ratios (HR) and 95% CIs were adopted as indicators of PFS and OS. HR and 95% CIs were extracted directly from included articles. For articles containing only Kaplan–Meier curves, survival data was extracted indirectly using Engauge Digitizer version 12.1, and outcomes were then derived pursuant to methods proposed by Tierney et al. [24] Pooled HR was calculated using an inverse variance-weighted method. OR and HR < 1 denoted outcome that was greater in the adjuvant RT cohort. Fixed-effects or random-effects models were used depending on the study nature and statistical heterogeneity of included studies in each analysis.

Heterogeneity between studies were assessed using the Cochrane Q test and the Higgins I^2^ statics. If *p *value of the Cochrane Q was less than 0.1 or I^2^ value was larger than 50%, the presence of significant heterogeneity among studies was considered and a random-effects model was used. Otherwise, a fixed-effects model was employed. The risk of publication bias was evaluated statistically by calculation of the *p *value (two-sided) for Egger’s linear regression test and Begg rank correlation, and graphically by inspection of funnel plots. *p* values of > 0.05 in Egger’s test and Begg rank correlation test were considered as absence of significant publication bias. All statistical analyses were performed using R version 4.0.3 (R Foundation for Statistical Computing, Vienna, Austria, 2008) and the meta package [25].

## Results

### Literature search and study quality assessment

**Fig. 1:**
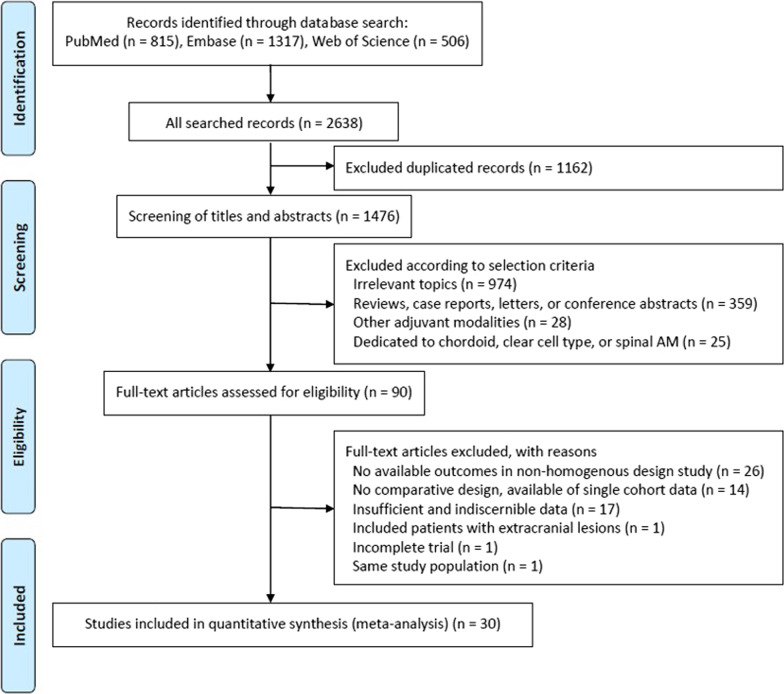
Flow diagram showing the selection of relevant studies

The initial search identified 815 studies in PubMed, 1317 in Embase, and 506 in Web of Science (Fig. 1). A total of 1162 duplicate studies and 1386 studies were excluded according to inclusion and exclusion criteria after reviewing titles and abstracts. The remaining 90 studies were subsequently given a full-text review. Of them, 60 studies were excluded because available data were not extractable in studies with non-homogenous design (n = 26), lack of comparative study arm (n = 14), insufficient and indiscernible data (n = 17), patients with extracranial lesions (n = 1), incomplete trial (n = 1), and overlapping study population (n = 1). Finally, a total of 30 studies were included in our meta-analysis (Table 1). Among these 30 studies included in the current meta-analysis, five had a homogenous study design, solely reporting the outcome of AM patients who underwent GTR. Twenty-five studies had a non-homogenous design, reporting about AM with STR (n = 22) or malignant meningioma (n = 3) as well as AM with GTR. All studies with a non-homogenous design contained extractable data for clearly defined outcomes, explicitly comparing observation and adjuvant RT after GTR for AM. All included studies had a retrospective design involving 2904 patients: 2167 were treated with GTR alone and 737 received adjuvant RT after GTR. GTR was defined as Simpson grade I-III in 19 studies, grade I-II in five studies, and grade I in three studies. Three studies described GTR or TR without using the Simpson grade.

**Table 1:** Baseline characteristics of included studies

References	Country	Design	Study period	WHO criteria	Simpson grade	No. of patients	
						Adjuvant RT	Observation
Condra et al. [28]	USA	R, OS, 1 institution	1964–1992	1993	1,2,3	4	21
Aghi et al. [44]	USA	R, OS, 1 institution	1993–2004	NR	1	8	100
Jo et al. [29]	Korea	R, OS, 1 institution	1997–2008	2000	1	19	13
Yu et al. [43]	China	R, OS, 1 institution	2003–2008	NR	1,2,3	47	11
Komotar et al. [9]	USA	R, OS, 1 institution	1992–2011	1993	1,2	13	32
Lee et al. [30]	USA	R, OS, 1 institution	1999–2009	2007	1,2,3	17	54
Park et al. [10]	Korea	R, OS, 1 institution	1997–2011	2000/2007	1,2,3	17	38
Aizer et al. [11]	USA	R, OS, 1 institution	1997–2011	NR	1,2,3	18	50
Sun et al. [45]	USA	R, OS, 1 institution	1993–2012	2007	1,2,3	39	112
Yoon et al. [12]	USA	R, OS, 2 institutions	2000–2010	2000	1,2,3	7	102
Wang et al. [26]	Taiwan	R, OS, 1 institution	2001–2009	2007	GTR	3	11
Jenkinson et al. [7]	UK	R, OS, 3 institutions	2001–2010	2000/2007	1,2,3	32	81
Yip et al. [32]	Taiwan	R, OS, 1 institution	2005–2014	NR	TR	7	16
Endo et al. [31]	Japan	R, OS, 1 institution	2000–2013	2007	1,2	11	19
Bagshaw et al. [13]	USA	R, OS, 1 institution	1991–2014	2007	1,2,3	12	40
Graffeo et al. [14]	USA	R, OS, 1 institution	1988–2011	2016	1,2,3	8	61
Dohm et al. [34]	USA	R, OS, 1 institution	1993–2014	2007	1,2,3	12	37
Alghamdi et al. [3]	Canada	R, OS, 1 institution	2003–2013	NR	GTR	1	43
Cho et al. [33]	Korea	R, OS, 1 institution	2003–2014	2000/2007	1,2	13	16
Shakir et al. [36]	Canada	R, OS, 1 institution	1992–2013	2007	1,2,3	12	28
Chen et al. [15]	USA	R, OS, 1 institution	1993–2014	1993/2000/2007	1,2,3	10	104
Budohoski et al. [35]	UK	R, OS, 3 institutions	2007–2014	2016	1,2,3	35	108
Zeng et al. [40]	China	R, OS, SEER data	2008–2015	2007	1,2,3	194	533
Zhi et al. [41]	USA	R, OS, 1 institution	2000–2012	2000/2007	1,2,3	26	72
Wang et al. [39]	USA	R, OS, 1 institution	2009–2018	2016	1,2,3	71	142
Lee et al. [16]	Korea	R, OS, 1 institution	2000–2013	2007	1,2,3	20	24
Ros-Sanjuan et al. [38]	Spain	R, OS, 1 institution	1994–2014	2016	1	5	5
Li et al. [37]	China	R, OS, 1 institution	2008–2015	NR	1,2	50	151
Lee et al. [42]	USA	R, OS, 1 institution	2000–2015	2000/2007	1,2,3	18	133
Jiang 2020 [27]	China	R, OS, 1 institution	2008–2016	2007	1,2	8	10

R, retrospective; OS, observational study; WHO, World Health Organization; GTR, gross total resection; RT, radiotherapy; SEER, Surveillance, Epidemiology, and End Results; NR, not reported

Qualitative assessment of selected studies is summarized in Additional file 1: Table S2. The risk of bias in the selection of participants was high in two studies that included AM patients located only in the skull base [26] and lateral ventricle [27]. Twenty-five studies with a non-homogenous design had a high risk of confounding bias because confounding variables were not considered between the adjuvant RT or the observation cohort after GTR [3, 7, 10, 12, 13, 15, 16, 26–42]. One study with a non-homogenous design that adjusted for major confounding variables using propensity score matching was considered to have a low risk of confounding bias [11]. One study with a homogenous study design had a high risk of confounding bias because of insufficient information for confounding variables [43]. The risk of performance bias due to an inadequate measurement of intervention was high in seven studies without a clear description of the definition of adjuvant RT [7, 11, 12, 14, 15, 29, 44]. The detection bias was low in all included studies. The attrition bias was high in 11 studies with missing data because of restriction in follow-up duration or loss to follow-up for analyzing the outcome [3, 10, 30, 35, 36, 39–43, 45]. The risk of reporting bias was high in five studies that did not report the recurrence number or the percentage of each cohort [26, 34, 37, 40, 41].

### Local recurrence

**Fig. 2:**
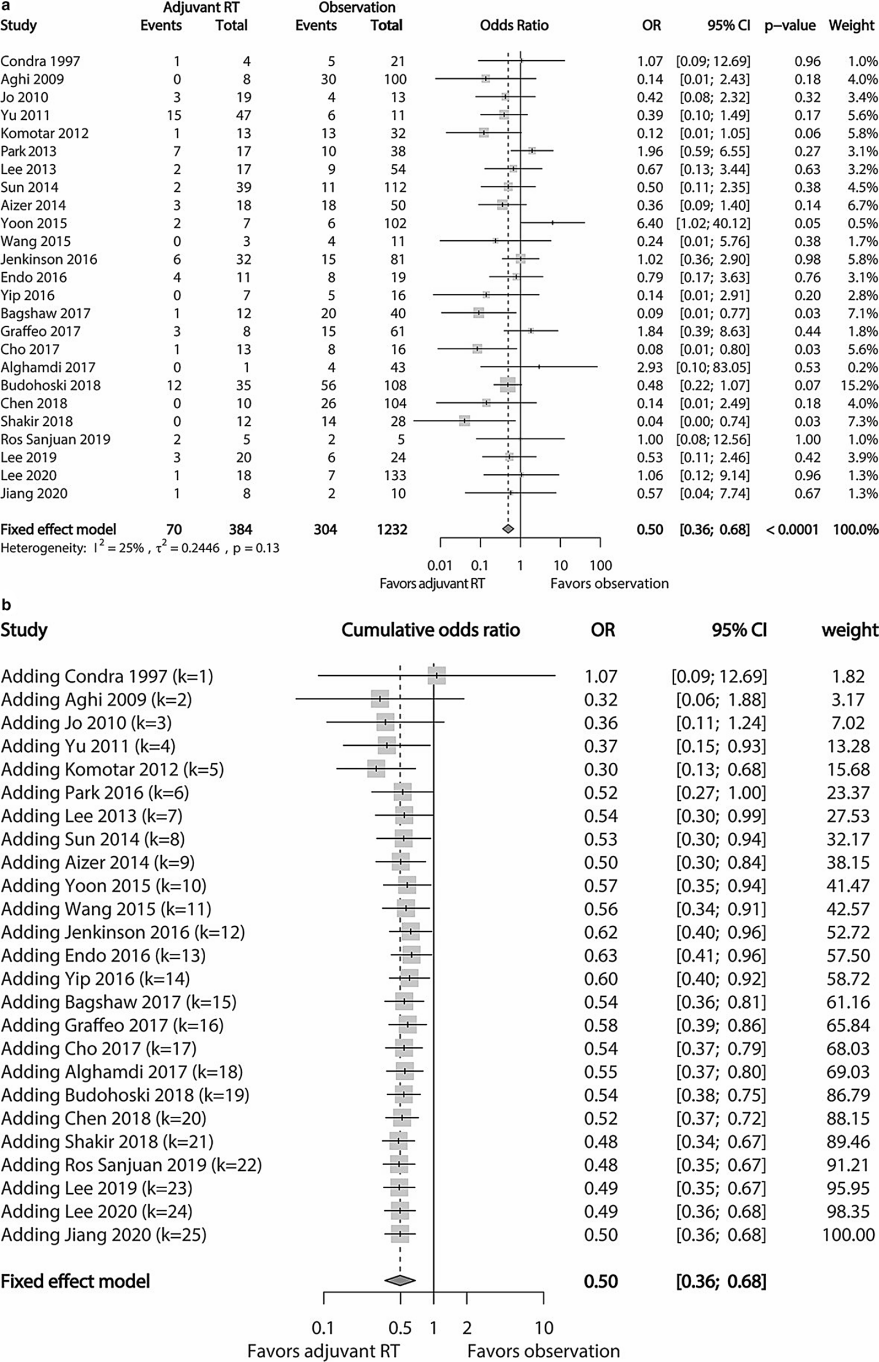
**a** Forest plot comparing the risk of local recurrence between the adjuvant RT cohort and the observation cohort. The risk of local recurrence is higher in the observation cohort. **b** Cumulative forest plot showing pooled OR of local recurrence according to the publication year. The first row shows the effect based on one study. The second row shows the cumulative effect based on two studies, and so on

Of 30 studies included in our meta-analysis, 25 studies consisting of 1232 patients in the observation cohort and 384 in the adjuvant RT cohort reported relevant data regarding local recurrence after GTR for AM. The crude local recurrence rate in the adjuvant RT cohort was 18.23% (70/384), which was statistically lower than that (24.68%, 304/1232) of the observation cohort (*p* = 0.009). Subsequent pooled analysis of these studies revealed that adjuvant RT reduced the risk of local recurrence (OR 0.50; 95% CI 0.36–0.68; *p* < 0.0001) (Fig. 2a). Included studies had no significant heterogeneity (I^2^ = 25%; Tau^2^ = 0.2446; *p* = 0.13). Cumulative analysis according to the publication year of each study showed that the adjuvant RT cohort had a benefit in controlling local recurrence over the observation cohort, showing statistical significance in recent years (Fig. 2b).

**Fig. 3:**
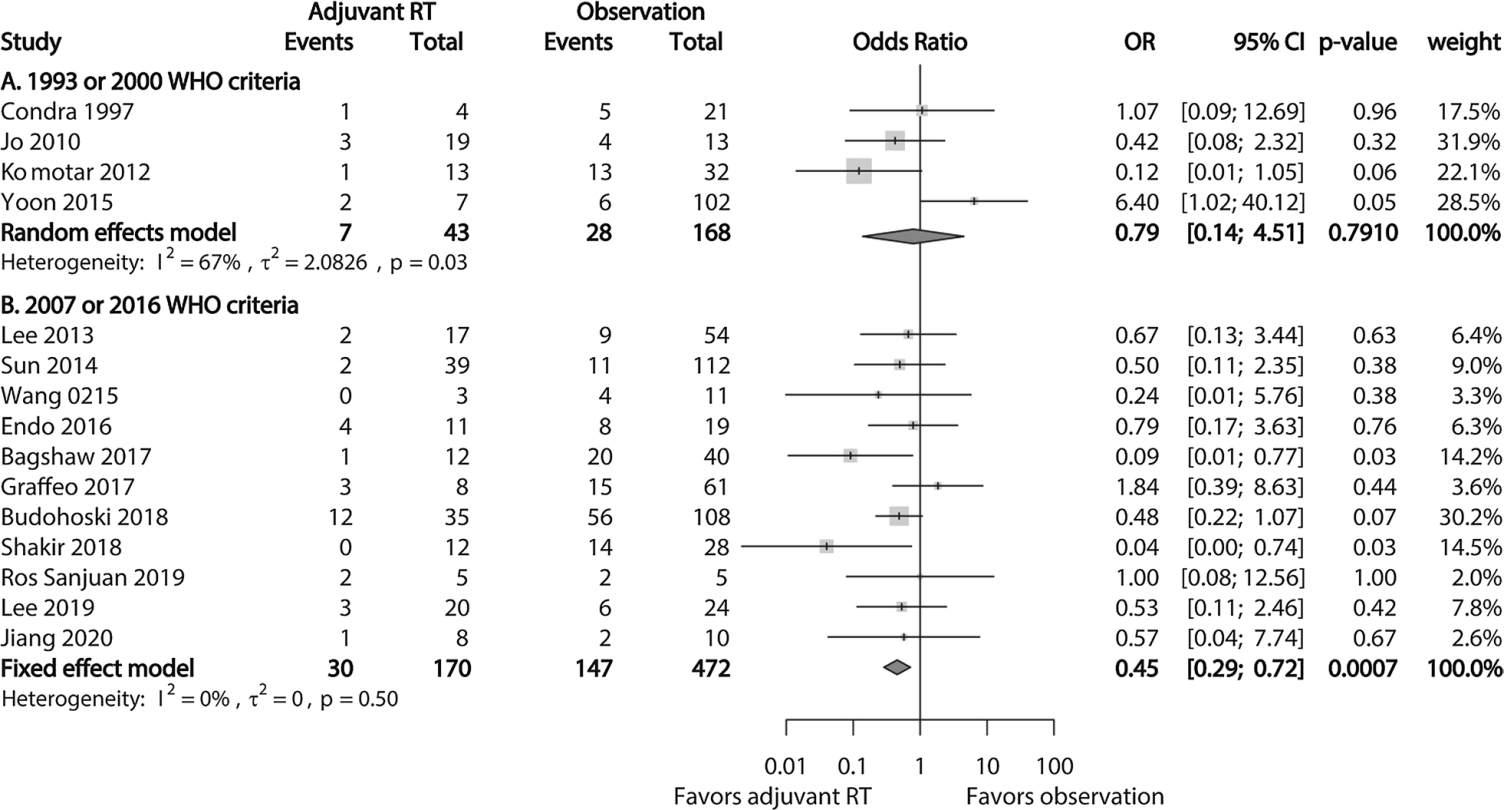
Forest plots comparing the risk of local recurrence between the adjuvant RT cohort and the observation cohort in subgroups using **a** 1993 or 2000 WHO criteria and **b** 2007 or 2016 WHO criteria for included studies. The benefit of adjuvant RT in local recurrence is significantly higher in a subgroup using the 2007 or 2016 WHO criteria

A subgroup analysis based on the WHO classification applied in each study showed that the crude local recurrence rate in studies using the 2007 or 2016 WHO classification was 17.65% (30/170) in the adjuvant RT cohort, which was significantly better than that (31.14%, 147/472) in the observation cohort (*p* = 0.001). Pooled analysis of studies of this subgroup showed significant advantage of adjuvant RT compared to observation in terms of local recurrence of AM patients after GTR (OR 0.45; 95% CI 0.29–0.72; *p* = 0.0007) (Fig. 3b). Included studies had no significant heterogeneity (I^2^ = 0%; Tau^2^ = 0; *p* = 0.50). In subgroup analysis of studies using the 1993 or 2000 WHO classification, there was no significant difference in crude local recurrence rate between the observation cohort (16.67%) and the adjuvant RT cohort (16.28%; *p* = 0.951). Pooled analysis of corresponding studies showed no significant difference in local recurrence between the adjuvant RT cohort and the observation cohort (OR 0.79; 95% CI 0.14–4.51; *p* = 0.7910) (Fig. 3a). A random-effect model was applied because of significant heterogeneity (I^2^ = 67%; Tau^2^ = 2.0826; *p* = 0.03) among studies in this subgroup.

### Survival outcomes

**Fig. 4:**
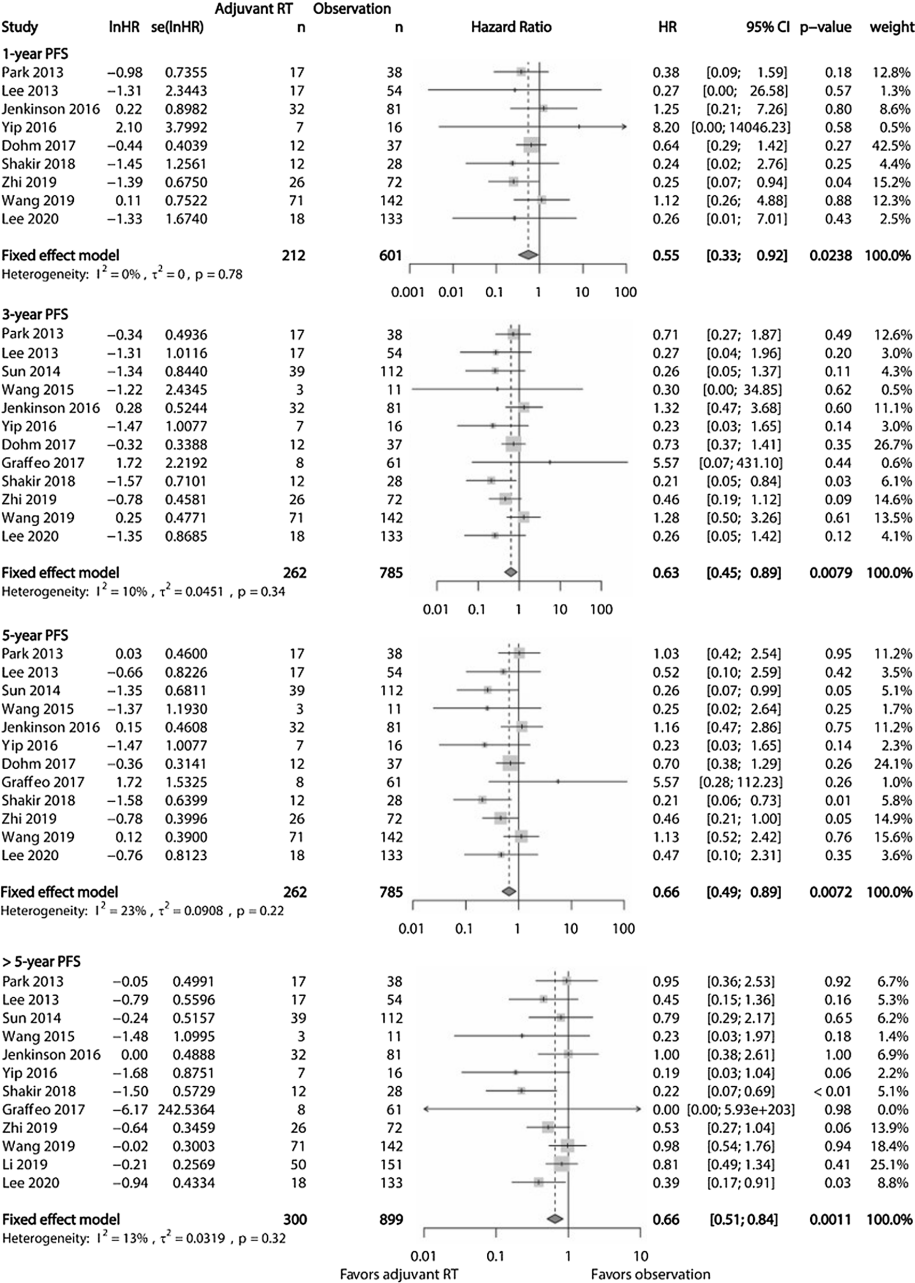
Forest plots showing PFS at each period of 1-year, 3-year, 5-year, and > 5-year between the adjuvant RT cohort and the observation cohort. The advantage of PFS is observed in the adjuvant RT cohort, showing increasing significance with longer period

**Fig. 5:**
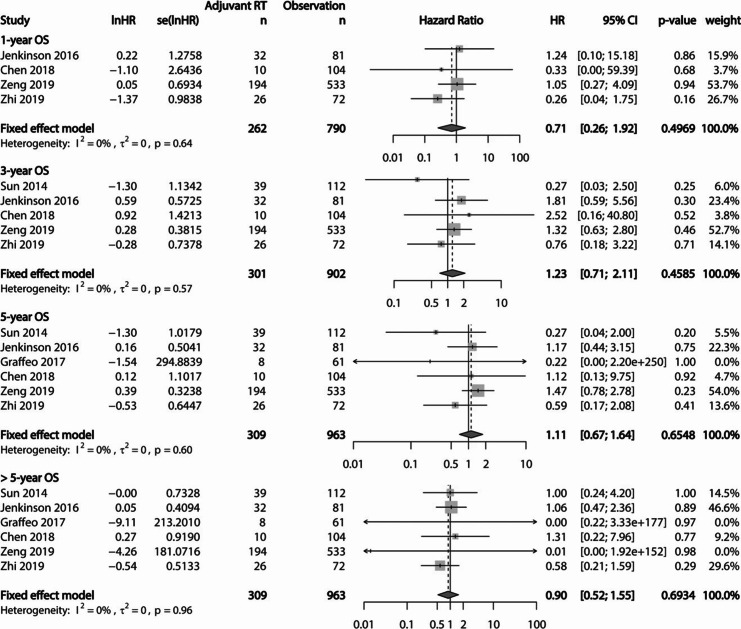
Forest plots showing OS at each period of 1-year, 3-year, 5-year, and > 5-year between the adjuvant RT cohort and the observation cohort. There was no significant difference in pooled HR of OS between the two cohorts

PFS and OS were evaluated at each period of 1-year, 3-year, 5-year, and > 5-year. Serially pooled HRs of PFS revealed that adjuvant RT was superior to observation after GTR for AM, showing sustained significance with a long-term follow-up (Fig. 4). Actuarial 1, 3, and 5-year PFS rates were 93.4%, 84.7%, and 80.5%, respectively, in the adjuvant RT cohort. These rates were 88.8%, 77.8%, and 68.3%, respectively, in the observation cohort. However, pooled HR of OS showed no significant differences between the adjuvant RT cohort and the observation cohort after GTR for AM (Fig. 5). Actuarial 1, 3, and 5-year OSs rate were 96.8%, 90.6%, and 86.1%, respectively, in the adjuvant RT cohort. These rates were 96.1%, 91.6%, and 86.6%, respectively, in the observation cohort.

### Toxicities of Radiation

Adverse events related to RT were reported in 13 studies. Information on radiotherapy and adverse events of each study based on CTCAE 5.0 are presented in Table 2. There was no documented event of Grade 5 toxicity. Most toxicities were consistent with Grade 1 or 2. Grade 3 and 4 toxicities were reported in 7 (2.1%) and 9 patients (2.7%), respectively.

**Table 2:** Toxicities of radiotherapy

Study	No. of patients	Radiation technology	Radiation dose (Gy)			Adverse events according to CTCAE grade (No. of patients)
			Median	Range	Per fx (no. of fx)	
Aghi et al. [44]	8	NR	60.2^†^	59.4–61.2	1.5–1.8	Grade 4 radiation necrosis (n = 1)
Komotar et al. [9]	13	NR	59.4	NR	1.8–2.0	Grade 1–2 skin dermatitis, erythema, alopecia, fatigue, and headache (NR)
Park et al. [10]	17 + 10*	Conventional RT/ 3D-CRT	61.2	40.0–61.2	NR (30–35)	Grade 1–2 fatigue, headache, nausea, dizziness, skin irritation (NR)
Aizer et al. [11]	18 + 12*	NR	60.0	55.8–64.0	NR	Grade 4 radiation related glioblastoma (n = 1)
Sun et al. [45]	39	NR	53.8	46.0–60.0	1.7–2.0	Grade 1–2 radiation necrosis (n = 1) Grade 4 radiation necrosis (n = 3)
Wang et al. [26]	3 + 9*	NR	NR	54.0–60.0	NR (27–30)	Grade 1–2 headache, dizziness, and skin irritation (NR)
Bagshaw et al. [13]	12 + 9*	NR	54.0	45.0–59.4	NR	Grade 1 fatigue, headache, and seizure (n = 4) Grade 2 headache, dizziness, alopecia, and hearing and memory impairment (n = 4) Grade 3 radiation necrosis (n = 2) Grade 4 optic nerve disorder (n = 1)
Graffeo et al. [14]	8	NR	54.0	50.5–61.2	NR (28–30)	Undetermined grade major morbidity (n = 1)
Dohm et al. [34]	12 + 51*	3D-CRT	55.7	50.4–59.4	NR (28–35)	Grade 4 seizure (n = 1) Grade 3 radiation necrosis, cognitive disturbance, peripheral neuropathy, seizure, aphasia, and optic nerve disorders (n = 7)
Cho et al. [33]	13 + 21*	Conventional RT	NR	30.0–61.2	NR (30–35)	Grade 1–2 fatigue, headache, nausea, dizziness, and skin irritation (NR)
Shakir et al. [36]	12 + 3*	3D-CRT	54.0	52.2–59.4	1.8	Grade 1–2 headache, dizziness, and paresthesia (n = 8)
Lee et al. [16]	20 + 33*	3D-CRT/IMRT	50.4/54.0	36.0–64.0/50.4–59.4	1.8–2.0	Grade 1 radiation necrosis (n = 2)
Ros-Sanjuan et al. [38]	5 + 8*	3D-CRT	NR	NR	NR	Undetermined grade radiation necrosis (n = 1)

CTCAE, Common Terminology Criteria for Adverse Events; RT, radiotherapy; 3D-CRT, three-dimensional conformal radiotherapy; IMRT, intensity-modulated radiotherapy; NR, not reported

*Patients no. such as AM with STR or malignant meningioma who underwent RT in studies with non-homogenous design

^†^Indicate mean value

### Publication bias

Publication bias results of included studies are shown in Table 3. All results of Egger’s test and Begg rank correlation had *p* value > 0.5 with relatively symmetric funnel plots, indicating no substantial evidence for publication bias in the dataset.

**Table 3:** Summary of the publication bias in each meta-analysis

Evaluation index		Egger’s linear regression test					Begg rank correlation		
		Intercept	SE	95% CIs	t value	*p* value	Tau	z value	*p *value
LR	Overall	− 0.839	0.590	− 2.060 to 0.382	1.422	0.169	− 0.197	1.378	0.168
	1993/2000 WHO criteria	− 1.911	7.191	− 32.852 to 29.031	0.266	0.815	0	0	1.000
	2007/2016 WHO criteria	− 0.626	0.727	− 2.271 to 1.018	0.862	0.411	− 0.255	1.090	0.276
PFS	1-year	− 0.080	0.546	− 1.371 to 1.211	0.147	0.888	0.139	0.521	0.602
	3-year	− 0.794	0.675	− 2.298 to 0.710	1.177	0.267	− 0.076	0.343	0.732
	5-year	− 0.791	0.815	− 2.607 to 1.024	0.971	0.354	− 0.106	0.480	0.631
	> 5-year	− 1.094	0.662	− 2.570 to 0.382	1.652	0.130	− 0.409	1.851	0.064
OS	1-year	− 0.519	1.114	− 5.314 to 4.276	0.466	0.687	− 0.167	0.340	0.734
	3-year	− 0.668	0.969	− 3.752 to 2.416	0.689	0.540	− 0.100	0.245	0.807
	5-year	− 0.737	0.588	− 2.371 to 0.896	1.253	0.279	− 0.400	1.127	0.260
	> 5-year	0.026	0.338	− 0.913 to 0.965	0.077	0.942	− 0.267	0.751	0.452

LR, local recurrence; PFS, progression-free survival; OS, overall survival; WHO, World Health Organization; SE, standard error; CIs, confidence intervals

## Discussion

The current meta-analysis included 30 studies with 2167 patients in the observation cohort and 737 patients in the adjuvant RT cohort. The local recurrence rate was lower in the adjuvant RT cohort than that in the observation cohort. However, in subgroup analysis, this result was proven to be true for studies using the 2007 or 2016 WHO classification, but not for those adopting previous classifications. PFS was better in the adjuvant RT cohort than in the observation cohort regardless of the follow-up term. However, OS did not differ significantly between the two cohorts.

The impact of adjuvant RT following GTR on AM patients compared to observation remains controversial. Management strategies among physicians also vary in clinical practice due to conflicting results of previous individual studies [46]. A past meta-analysis has reported that postoperative RT may decrease the risk of tumor recurrence for AM patients after GTR [47]. However, it is controversial to determine the effectiveness of adjuvant RT compared to observation based on this single meta-analysis. It included only 14 studies published by 2012 with a relatively small number of total subjects (n = 757). Additionally, it included several studies with single-arm design which had an innate bias in assessing outcomes of relative values. Many studies have been reported since then. Outcomes of these studies should be considered for analyzing the benefit of adjuvant RT over observation following GTR for AM patients. We conducted an up-to-date and systematic review and meta-analysis involving 2167 patients in the observation cohort and 737 in the adjuvant RT cohort. In the present meta-analysis, we found that adjuvant RT had a benefit of decreasing local recurrence rate (OR 0.50; *p* < 0.0001) compared to observation for AM patients after GTR. Furthermore, the pooled outcome of prolonged PFS (HR: 0.67; *p* = 0.0024) also showed a high statistical significance that individual studies failed to confirm due to their small sample sizes. Results of the current study can help us establish appropriate management strategy for AM patients following GTR in clinical practice.

In general, brain invasion had long been recognized as an adverse factor that is related to a higher risk of local recurrence [44, 45, 48–50]. To reflect this widespread conception, the 2007 WHO classification first considered brain invasion as a staging feature in the diagnosis of AM. In the 2016 WHO classification, brain invasion was eventually included as a criterion that was sufficient for diagnosing AM by itself [2]. In our subgroup analysis of studies using the 2007 or 2016 WHO classification, adjuvant RT significantly reduced local recurrence (OR 0.45; *p* = 0.0007) with a low recurrence rate (17.65%) compare to observation (31.14%) for AM patients after GTR. This result implies the utility of adjuvant RT for AM patients after GTR, especially in the current era of the 2016 WHO classification when the diagnosis of AM is increasing. Unlike the subgroup analysis of studies using the 2007 or 2016 WHO classification, the subgroup analysis of studies on patients with AM according to the 1993 or 2000 WHO classification which did not include brain invasion as a diagnostic feature showed no significant difference in local recurrence rate between adjuvant RT and observation cohorts. Results of current meta-analyses are emphatic about the need of integrative assessment of brain invasion for higher accuracy using multimodalities such as histopathological, operative, and image findings for establishing appropriate treatment for AM patients.

In the current meta-analysis, there was no statistical difference in pooled OS for any period between adjuvant RT and observation cohorts, in line with results of included individual studies [39, 40]. Zeng et al. have reported that the OS of AM patients who undergo GTR only is similar to that of patients who receive adjuvant RT after GTR or STR regardless of the extent of resection [40]. The extent of resection for AM can be an overwhelmingly significant factor to improve the OS, enough to conceal the effect of adjuvant RT. The improvement of salvageable modalities for recurrent AM might have mitigated the difference of OS between two cohorts. Several studies have reported the benefit of survival extension through salvage therapy using ion radiotherapy, brachytherapy, and radiosurgery [51–53]. Despite the benefit of salvage therapy, aggressive upfront treatment after the first surgery for AM should be considered because of a low durability in local control of salvage therapy [51]. Furthermore, recurrent meningioma tends to be more aggressive than the original tumor, causing failure of salvage therapy. It may offset the effect of adjuvant RT on OS of AM patients [28, 54, 55].

Neurotoxicity of adjuvant RT is a major cause that makes physicians hesitate to apply it for AM patients after GTR. The incidence of neurotoxicity varies, ranging from 3.4% to 16.7% according to the location of the lesion, radiation dose, and radiation modality. However, advanced techniques for RT can lead to improvement in side effect profile and conventional fractionation RT can provide a far lower toxicity than hypofractionation RT or radiosurgery [56, 57]. Furthermore, most neurotoxicities are within tolerable levels using proper medical treatment [58]. In a phase II parallel observation study (EORTC 22,042–26,042), Weber et al. have reported that the rate of late adverse effect of CTCAE grade 3 or more associated with adjuvant RT following GTR for AM is 14.3% (3/56) and that there is no toxic death (grade 5) [18]. In studies included in the current review, no death related to RT was reported and the occurrence of toxicities of grade 3 or more was 5.4% (18/336). Documented grade 4 toxicities included radiation necrosis (n = 4), optic nerve disorder (n = 1), seizure (n = 1), and radiation-related glioblastoma (n = 1). Although toxicities are usually mild, serious toxicity such as optic neuropathy and radiation-induced malignancy should not be overlooked when considering adjuvant RT for AM patients after GTR. Therefore, it is imperative to carefully adjust the radiation dose and elaborate radiation technique to maximize the efficacy and minimize the toxicity in accordance with each patient. Additionally, although the toxicity of adjuvant RT is at low-level, risk factors for recurrence in AM patients after GTR should be considered to avoid unnecessary toxicity and select proper candidates of adjuvant RT. Chen et al. have reported that AM with MIB labelling index ≤ 7% have a very low risk (RR 0.06; 95% CI 0.003–0.32; *p* = 0.0004) of recurrence after GTR [15]. Tumor size, secondary AM, and bone involvement have been reported in prior studies as risk factors of recurrence for AM patients after GTR [15, 39].

Several limitations exist in the present meta-analysis. First and foremost, it should be noted that all studies included were retrospective in nature, thus limiting implications of results. Another up-to-date meta-analysis with the strongest level of evidence using two randomized phase II trials (ROAM/EORTC-1308 and NRG-BN003) will be performed in the future after these trials are completed. Second, like all meta-analyses, pooled results need to be interpreted acknowledging that local recurrence and survival outcomes can vary according to individual patients. Third, because of an insufficient number of studies with a homogenous design solely reporting outcomes of AM with GTR, we included studies with a non-homogenous design which might have caused confounding bias due to the fact that individual studies did not report confounding variables such as age, sex, location of the mass, or follow-up period according to the two cohorts of our interest. To reduce this risk of bias and provide an objective evaluation, we conducted meta-analyses using only dataset of direct comparative studies. Moreover, heterogeneities of most pooled analyses were low and acceptable except in analysis about local recurrence of studies using the 1993 or 2000 WHO criteria showing significant heterogeneity. Thus, the current meta-analyses about local recurrence and survival outcome have significant implications about the effect of adjuvant RT after GTR on AM patients compared to observation after GTR. Finally, the systematic review of toxicity should be interpreted with caution because it included adverse effects occurring in patient populations not strictly confined to those with AM and received adjuvant RT after GTR.

## Conclusions

Currently, available evidence supports the finding that adjuvant RT confers significant benefits for local control and PFS compared to observation in AM patients after GTR. However, adjuvant RT should be carefully considered to avoid unforeseen complications related to RT. It should be performed for AM patients with a high risk of recurrence after GTR. Randomized controlled trials are necessary to provide further evidence for our results and assess which AM patients could have the greatest clinical benefit and the lowest toxicity of adjuvant RT following GTR.

## Supplementary Information

**Additional file 1:** Supplementary tables for search strategies, quality assessment of the studies by RoBANS, and PRISMA 2009 Checklists.

## Data Availability

All data generated or analyzed during this study are included in this manuscript.
